# Management of neurotrophic corneal ulcer with a topical liposomal ozonated oil: A report on three clinical cases

**DOI:** 10.1016/j.ajoc.2025.102344

**Published:** 2025-04-29

**Authors:** Isabel Mogollón Giralt, Ana Boto de los Bueis, Ana Martín Ucero, Paola Vázquez Colomo

**Affiliations:** a Unidad de Cirugía Refractiva. Clínica Baviera, Calle Beatas 6, 288001, Alcalá de Henares, Spain; b Unidad de Superficie Ocular y Córnea, Hospital Universitario la Paz, Paseo de la Castellana 261, 28046 Madrid, Spain

**Keywords:** Corneal ulcer, Healing corneal wounds, Infectious keratitis, Neurotrophic ulcer, Liposomal ozonated oil

## Abstract

**Purpose:**

To present three cases of neurotrophic corneal ulcer successfully treated with topical liposomal ozonated oil (Ozonest®) eye drops and to assess its potential usefulness in preventing corneal infection and promoting epithelialization.

**Observations:**

Neurotrophic corneal ulcers require appropriate therapy that includes re-epithelialization and prevention of infection usually with antibiotic therapy. The emergence of multidrug-resistant bacterial strains due to the overuse of antibiotics in the treatment of infectious diseases has prompted researchers to investigate new antimicrobial formulations. Liposomal ozonated oil has demonstrated antiseptic (antibacterial, antifungal and antiviral) and tissue repair properties, making it a promising candidate for the prevention and treatment of acute and chronic local infections. Additionally, its specific formulation ensures good tolerability and biocompatibility with the ocular surface. In the three cases of neurotrophic ulcer presented here, topical liposomal ozonated oil (Ozonest®) was used, resulting in good resolution and complete re-epithelialization without superinfection.

**Conclusions and importance:**

The use of topical liposomal ozonated oil (Ozonest®) may have an antiseptic role and help in the closure of neurotrophic ulcers.

## 1 Introduction

In healthy corneas, corneal epithelial cells undergo regeneration from limbal stem cells, exhibiting an average lifespan and self-renewal rate of 7–10 days.^1^ Consequently, an acute epithelial defect generally regenerates within a timeframe of 7–14 days.^2^^,^^3^ Conversely, persistent epithelial defects (PEDs) are defined as corneal defects with a minimum area of 2 mm^2^ that persist without improvement for more than two weeks despite conventional treatment.^4^ Neurotrophic keratopathy, a degenerative corneal epithelial disorder caused by compromised corneal innervation, is a form of PED in which an extensive range of conditions can lead to damage the trigeminal nerve at any level.^5^ A reduction in corneal sensitivity or complete corneal anesthesia is the defining characteristic of this disease, which can result in epithelial keratopathy, ulceration, and perforation.

The cornea contains a high density of nerve endings from the long posterior ciliary nerves, and these sensory neurons have been demonstrated to exert a direct influence on the integrity of the corneal epithelium. In the event of neuronal destruction, as observed in neurotrophic keratopathy, the epithelial cells undergo a series of changes, including swelling, loss of microvilli, and the production of an abnormal basal lamina.^6^, ^7^, ^8^ This may result in the delay or cessation of mitosis, which could subsequently lead to epithelial breakdown.^9^ The layers of non-keratinized stratified squamous epithelial cells and tight junctions of the corneal epithelium act as a barrier, preventing the entry of pathogenic organisms.^10^ Therefore, any violation of the corneal epithelium's continuity in an ulcer poses a risk of infection, which is increased in PEDs due to the longer exposure time. However, the probability of infection in PEDs or neurotrophic ulcers and the percentage of resistant infectious keratitis in these patients remain unknown.

Neurotrophic keratitis is classified into three stages, delineated by the severity of the corneal defect, according to the Mackie classification. Stage 1 (mild) is typified by a superficial punctate keratitis, stage 2 (moderate) is defined by a persistent epithelial defect, and stage 3 (severe) is characterized by a corneal ulcer with stromal lysis, which may result in perforation.^11^ In all of these stages, the absence of pain is attributed to a reduction in corneal sensitivity. Indeed, the diagnosis is based on the demonstration of reduced corneal sensitivity, which can be evaluated through the use of esthesiometry or with a cotton-tipped applicator.

The objective of treatment for neurotrophic keratopathy is to restore corneal integrity and prevent corneal infection. A variety of treatment modalities can be used, typically depending on the severity of the disease. The following treatments have been traditionally used to promote epithelial closure: topical (hydration with artificial tears and preservative-free gels or ointments), systemic (doxycycline with an anticollagenolytic effect), and mechanical (contact lens, amniotic membrane, palpebral closure). Recently, the use of insulin drops has been shown to facilitate the healing of neurotrophic epithelial defects.^12^ To prevent infection, antibiotic therapy has traditionally been used; however, the overuse of antibiotics and the use of inappropriate dosing regimens have contributed significantly to the development of antibiotic resistance.

In response to the growing challenge of antibiotic resistance, new antimicrobial agents with novel mechanisms of action have been developed. Among these, ozone has been successfully used in ophthalmology despite its instability and its damaging effect on cell wall and DNA.^13^, ^14^, ^15^, ^16^, ^17^, ^18^, ^19^, ^20^ By ozonating monosaturated fatty acids and encapsulating with hypromellose in liposomes to improve stability and tolerance on the ocular surface,^21^, ^22^, ^23^, ^24^ liposomal ozonated oil is increasingly being considered as a treatment option for various types of infections. The antimicrobial, antifungal and antiviral properties of liposomal ozonated oil are well documented, as its ability to prevent the development of antimicrobial resistance and allergic reactions.^20^^,^^23^^,^^25^, ^26^, ^27^, ^28^, ^29^, ^30^, ^31^, ^32^

In this case series we assess the potential utility of liposomal ozonated oil 0.5 % (Ozonest®, Esteve Pharmaceuticals SA, containing a 0.5 % self-preserved ozonated oil with hypromellose and 10 % liposomes) eye drops in the prevention of corneal infection and promotion of epithelialization in corneal ulcers of neurotrophic etiology. The fluorescein test was used to evaluate corneal defects, and a negative fluorescein test or lack of fluorescein staining indicates the absence of an epithelial defect.

## 2 Findings

### 2.1 Case 1

A 65-year-old female patient presented to the emergency room with intense redness and a sensation of a foreign body in her left eye. The patient had previously undergone pars plana vitrectomy surgery for a retinal detachment. Upon examination, the patient exhibited conjunctival hyperemia, dense leukoma in the lower periphery involving approximately one third of the cornea, and ulcers with overrolled edges in the temporal and nasal regions.

**Fig. 1:**
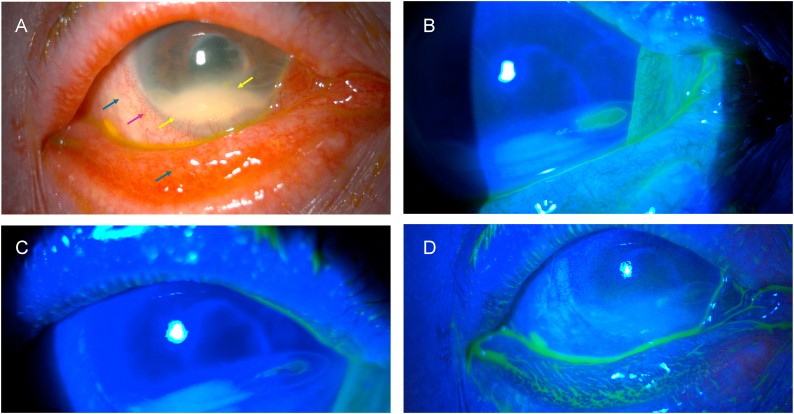
Left eye of a woman with secondary neurotrophic ulcer after retinal detachment surgery and stromal opacity in the inferior cornea. A) At initial diagnosis, conjunctival hyperemia (blue arrows), ciliary injection (purple arrow), dense stromal opacity (leukoma) (yellow arrows) in the lower periphery involving one third of the cornea and epithelial defect with overrolled edges in the temporal and nasal area are seen. B) Fluorescein shows staining of the nasal epithelial defect at initial diagnosis. C) After 1 week of treatment, the epithelial defects do not stain. D) After 1 month of treatment, epithelial defects remain closed (no staining). (For interpretation of the references to colour in this figure legend, the reader is referred to the Web version of this article.)

Baseline visual acuity after retinal detachment surgery and at the time of examination in the emergency room was limited to light perception in the affected eye. Corneal sensitivity assessed qualitatively with a cotton tip revealed complete anesthesia in all 4 quadrants of the cornea. Treatment was initiated with artificial tears containing hyaluronic acid, insulin eye drops 1 IU/mL (3 times daily) and liposomal ozonated oil eye drops (one drop, 4 times daily), along with the application of a therapeutic contact lens. After 7 days of treatment, which included both liposomal ozonated oil and the aforementioned adjuvant therapies, an initial improvement in the clinical condition was observed. This improvement was attributed to the onset of the epithelialization process, with a progressive reduction of the ulcer area and complete epithelialization after one month of treatment. Fig. 1 shows the mentioned findings in slit lamp examination at both the time of diagnosis and after one month of treatment.

### 2.2 Case 2

A 72-year-old female patient presented to the emergency room with a one-week history of intense redness and gritty feeling in her left eye. The patient had previously undergone retinal detachment surgery, including pars plana vitrectomy and cerclage, as well as an epiretinal membrane detachment surgery, treated by pars plana vitrectomy and peeling of the epiretinal and internal limiting membranes. These procedures had been performed in both eyes. Upon examination, the patient's visual acuity was limited to hand movements, with conjunctival hyperemia, corneal epithelial irregularities, an ulcer with overrolled edges in the inferior paracentral area. Additionally, corneal thinning and melting were present, along with stromal edema and cellularity in the anterior chamber. Corneal sensitivity was assessed qualitatively with a cotton tip, revealing complete anesthesia in all 4 quadrants of the cornea.

Oral therapy was initiated with doxycycline 100 mg once daily, along with topical therapy consisting of artificial tears with hyaluronic acid, insulin eye drops (3 times daily), liposomal ozonated oil eye drops (one drop, 4 times daily), and the application of a therapeutic contact lens. After 7 days of treatment, including both liposomal ozonated oil and the aforementioned adjuvant therapies, the clinical condition showed initial improvement, with reduction in the ulcer area and decreased cellularity in the anterior chamber.

**Fig. 2:**
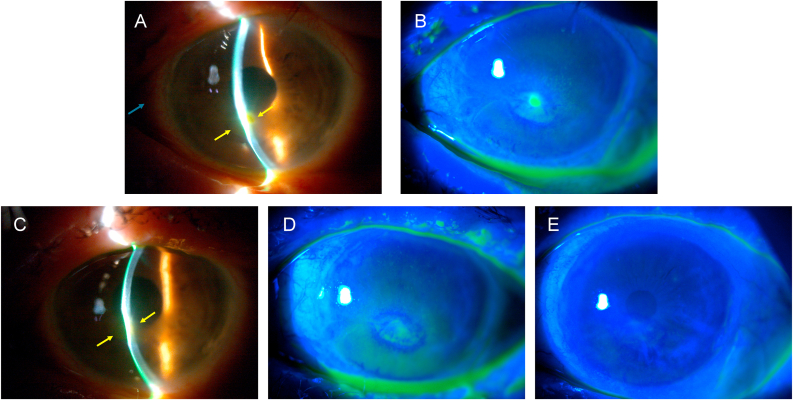
Left eye of a woman with secondary neurotrophic ulcer after retinal detachment and epiretinal membrane surgery. A) At initial diagnosis, conjunctival hyperemia (blue arrow), ciliary infection, corneal epithelial irregularity, epithelial defect with overrolled edges (yellow arrows) in the inferior paracentral area with corneal thinning and melting, stromal edema and cellularity in the anterior chamber are seen. B) Fluorescein shows staining of the epithelial defect at initial diagnosis. C) After 1 week, we still observe corneal epithelial irregularity, epithelial defect with overrolled edges (yellow arrows) in the inferior paracentral area with corneal thinning and melting but less stromal edema and cellularity in the anterior chamber. D) Fluorescein shows less staining of the epithelial defect after 1 week. E) After 1 month of treatment, resolution of the ulcer (no fluorescein staining), leaving slight thinning and faint leukoma. (For interpretation of the references to colour in this figure legend, the reader is referred to the Web version of this article.)

The patient continued treatment with liposomal ozonated oil (4 times daily), and after 14 days the cornea had begun to reepithelialize, with a decrease in corneal edema and disappearance of cellularity in the anterior chamber. One month later, restoration of epithelial integrity was achieved, confirmed by a negative fluorescein test. The patient experienced a gradual and progressive recovery of the corneal anatomy, with resolution of the ulcer, leaving only slight thinning and a faint leukoma (Fig. 2), reaching a visual acuity of 0.05 (equivalent to 20/400 in the Snellen chart).

### 2.3 Case 3

A 75-year-old male patient presented to the emergency room with a one-week history of intense redness and a foreign body sensation in his right eye. The patient had undergone trigeminal nerve surgery for trigeminal neuralgia one year earlier. Upon examination, the patient exhibited conjunctival hyperemia, a central corneal ulcer with overrolled edges measuring 1.5 mm × 4.5 mm, and diffuse superficial punctate keratopathy grade III.

The best corrected visual acuity was 0.36 (equivalent to 20/63 in the Snellen chart). The corneal sensitivity was evaluated qualitatively with a cotton tip, revealing complete anesthesia in the 4 quadrants of the cornea. Therapy was initiated with Oftalmowell (gramicidin, neomycin, polymyxin B) every 6 hours, artificial tears containing hyaluronic acid, a night ointment composed of vitamin A palmitate, liquid paraffin, and white soft paraffin, and the application of a therapeutic contact lens. After 7 days of treatment, the clinical condition showed improvement, with a reduction in the ulcer area measuring 0.2 mm × 1.5 mm. At this point, the patient was referred to the corneal clinic, where antibiotic treatment was discontinued and replaced by liposomal ozonated oil (one drop, 4 times daily) and insulin eye drops (every 8 hours).

**Fig. 3:**
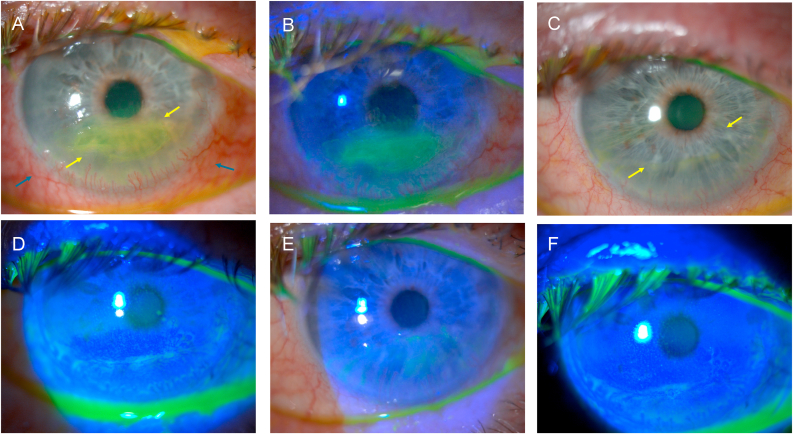
Right eye of a man with secondary neurotrophic ulcer after trigeminal nerve surgery. A) At initial diagnosis, conjunctival hyperemia (blue arrows), ulcer with overrolled edges (yellow arrows) measuring 1.5 mm × 4.5 mm in the central area and diffuse superficial punctate keratopathy grade III is seen. B) Fluorescein shows staining of the epithelial defect at initial diagnosis. C) After 1 week, reduction of the epithelial defect (yellow arrows) to 0.2 mm × 1.5 mm is seen. D) After 1 week, less fluorescein staining is seen. E) After 1 month, restoration of epithelial integrity is seen. F) After 1 month of treatment, no fluorescein staining is seen. (For interpretation of the references to colour in this figure legend, the reader is referred to the Web version of this article.)

One month later, the restoration of epithelial integrity was confirmed with a negative fluorescein test. A gradual, progressive improvement of the corneal anatomy was observed, with resolution of the ulcer, complete epithelialization, and no residual stromal opacity (Fig. 3) and best corrected visual acuity of 0.88 (equivalent to 20/25 in the Snellen chart).

## 3 Discussion

The main objective of the three cases presented here was to assess the management of neurotrophic corneal ulcers with liposomal ozonated oil.

Traditionally, broad-spectrum antibiotic therapy has been used to protect epithelial defects from infection in neurotrophic keratopathy, but this may have drawbacks, as it protects against certain bacteria, but does not protect against infection by viruses (sometimes implicated in the etiopathogenesis of neurotrophic keratopathy) and fungi (implicated in 27 % of corneal infections in therapeutic lens wearers),^33^ particularly in patients with PED. In addition, many of these antibiotic eye drops contain preservatives that are aggressive to the epithelium. Furthermore, it is well known that the use of broad-spectrum antibiotic therapy can lead to an increased risk of antimicrobial resistance.^34^ On the other hand, compared to antibiotic therapy, liposomal ozonated oil in contact with rapidly replicating microbial cells releases highly oxidative oxygenated compounds responsible for its antibacterial, antifungal, and antiviral action.^35^, ^36^, ^37^ It has also been shown to reduce ocular surface bacterial load comparable to 5 % povidone-iodine and superior to ofloxacin in dogs.^38^

The clinical efficacy of liposomal ozonated oil solutions against infections in ophthalmic patients is limited to a small number of cases, and without a control group, in the treatment of corneal ulcers, dendritic and traumatic keratitis, and as a preoperative measure in cataract surgery.^24^^,^^27^^,^^35^^,^^36^^,^^39^

In the three cases presented, despite the use of contact lenses, which predisposes to the risk of infection by aggressive bacteria and fungi, no patient suffered superinfection until approximately one month after the start of treatment with Ozonest, when the epithelial closure was achieved. This modest article suggests that Ozonest may avoid adding antibiotics in persistent epithelial defects, while preserving a more protective policy on antibiotic resistance.

Similarly, another fundamental axis of neurotrophic keratopathy management is the promotion of epithelial defect closure, for which topical (hydration with artificial tears, preservative-free gels or ointments and insulin drops), systemic (doxycycline with anticollagenolytic effect) and mechanical protection (contact lens, amniotic membrane, palpebral closure) treatments have traditionally been used.

In vitro studies have demonstrated that gaseous ozone, at concentrations of 0.5–2 ppm, can have a damaging effect on microorganisms, which also extends to the ocular surface, involving the epithelial and goblet cells and increasing the levels of inflammatory cytokines.^40^ However, 5 % liposomal ozonated oil, when administered as 1–2 drops 3–4 times daily, has been shown to have no adverse effects on the epithelial cells of the cornea and conjunctiva. Instead, it promotes cellular adaptation to oxidative stress by activating endogenous antioxidant and anti-inflammatory mechanisms.^18^^,^^41^^,^^42^ Recently, liposomal ozonated oil has been shown to promote wound healing and remodeling when applied to corneo-conjunctival tissues through collagen synthesis and stimulation of platelet-derived growth factor (PDGF) and transforming growth factor beta (TGFB) production.^18^^,^^41^, ^42^, ^43^, ^44^ It also activates immunity by increasing interferon, cytokines and antioxidant enzymes and enhances the immune response by regulating nitric oxide, NF-kB and TNF-alpha levels.^18^^,^^41^^,^^45^ In an animal model of Candida keratitis treated with ozonated distilled water and ozonated olive oil, these treatments showed greater efficacy than fluconazole or the control group. Clinical improvement was observed in eyes with keratitis, likely due to the microbicidal effect and its anti-inflammatory properties, which may involve the oxidation of prostaglandins.^30^

Liposomal ozonated oil has been successfully used in patients with corneal ulcers and keratitis,^39^ as well as an adjuvant therapy in external ocular pathologies such as vernal conjunctivitis and granulomatous conjunctivitis.^46^

Therefore, liposomal ozonated oil has demonstrated not only the absence of toxicity in human corneal and conjunctival cells but also regenerative and anti-inflammatory properties at therapeutic doses, which would contribute to one of the objectives sought in the treatment of neurotrophic keratopathy.

In the three cases presented here, epithelial closure was achieved in all patients within the first month of treatment. However, due to the severity of the neurotrophic ulcers in these patients (Mackie stages 2 and 3), they were treated with insulin eye drops, doxycycline, and contact lenses to promote re-epithelialization. The combination of these therapies acted as a confounding factor, making it difficult to evaluate the specific re-epithelializing effect of liposomal ozonated oil in vivo. The lack of a control group further limits the ability to determine whether the treatment itself accelerates corneal regeneration. To better assess this effect, future studies could focus in patients with Mackie stage 1 neurotrophic ulcers who do not require additional treatment.

Liposomal ozonated oil has been used in the treatment of several ophthalmic pathologies and has demonstrated its beneficial antimicrobial and restorative properties. These properties have been observed in conditions such as in conjunctivitis, keratitis, keratoconjunctivitis and corneal ulcers.^24^^,^^27^^,^^47^ Recently, liposomal ozonated oil has also shown efficacy in improving the signs and symptoms of blepharitis.^48^

## 4 Conclusions

Liposomal ozonated oil can be an innovative alternative in the treatment of neurotrophic keratopathy due to its antiseptic and regenerative effects. Its major advantage is the broad antimicrobial spectrum which may aid in preventing antibiotic resistance.

These encouraging results could make it a significant therapeutic alternative to the use of antibiotics. Due to the small number of patients, further studies are needed to confirm the efficacy and safety of this technique.

## CRediT authorship contribution statement

**Isabel Mogollón Giralt:** Writing – review & editing, Writing – original draft, Validation, Supervision, Resources, Project administration, Methodology, Investigation, Conceptualization. **Ana Boto de los Bueis:** Resources, Investigation. **Ana Martín Ucero:** Resources, Investigation. **Paola Vázquez Colomo:** Resources, Investigation.

## Patient consent

Consent to publish the case report was not obtained. This report does not contain any personal information that could lead to the identification of the patient.

## Funding

This study was supported by 10.13039/100017521Esteve Pharmaceuticals SA, who provided an unrestricted grant. Esteve Pharmaceuticals was not involved in the conception, collection, analysis, and interpretation of data, in the writing of the manuscript, or in the decision to publish the results.

## Declaration of competing interest

The authors declare that they have no known competing financial interests or personal relationships that could have appeared to influence the work reported in this paper.
